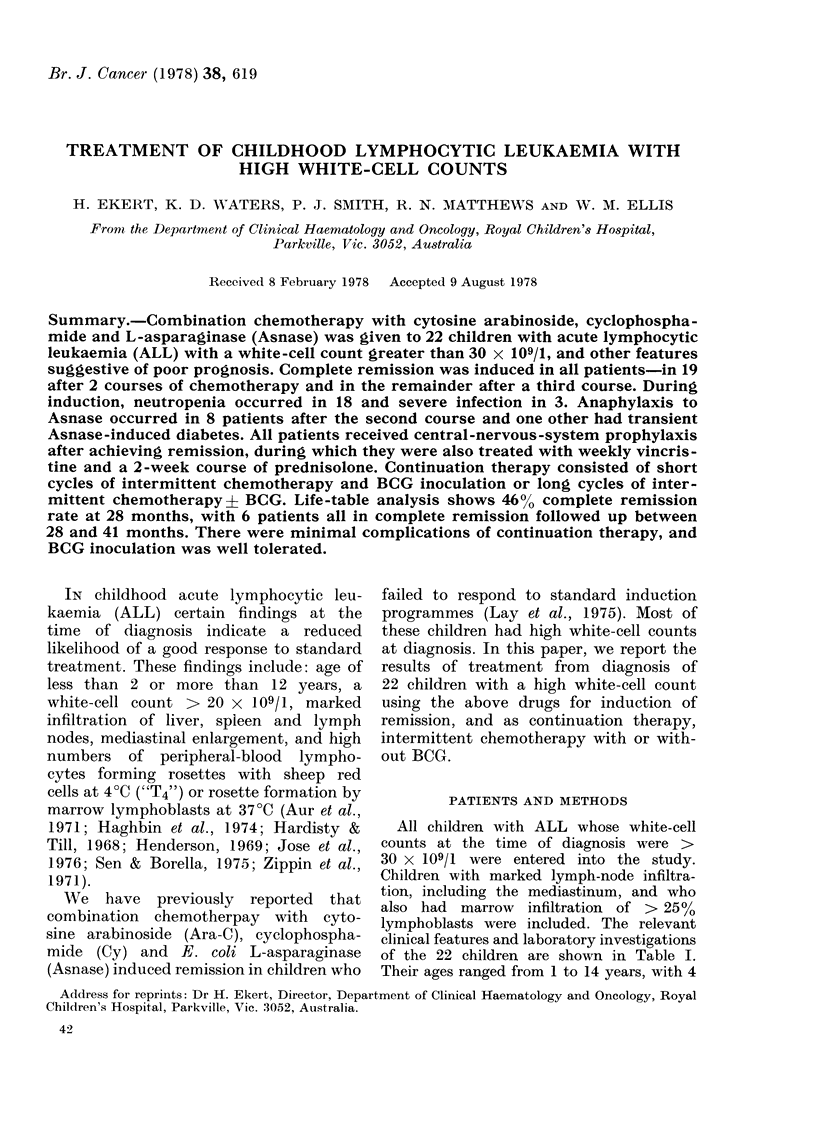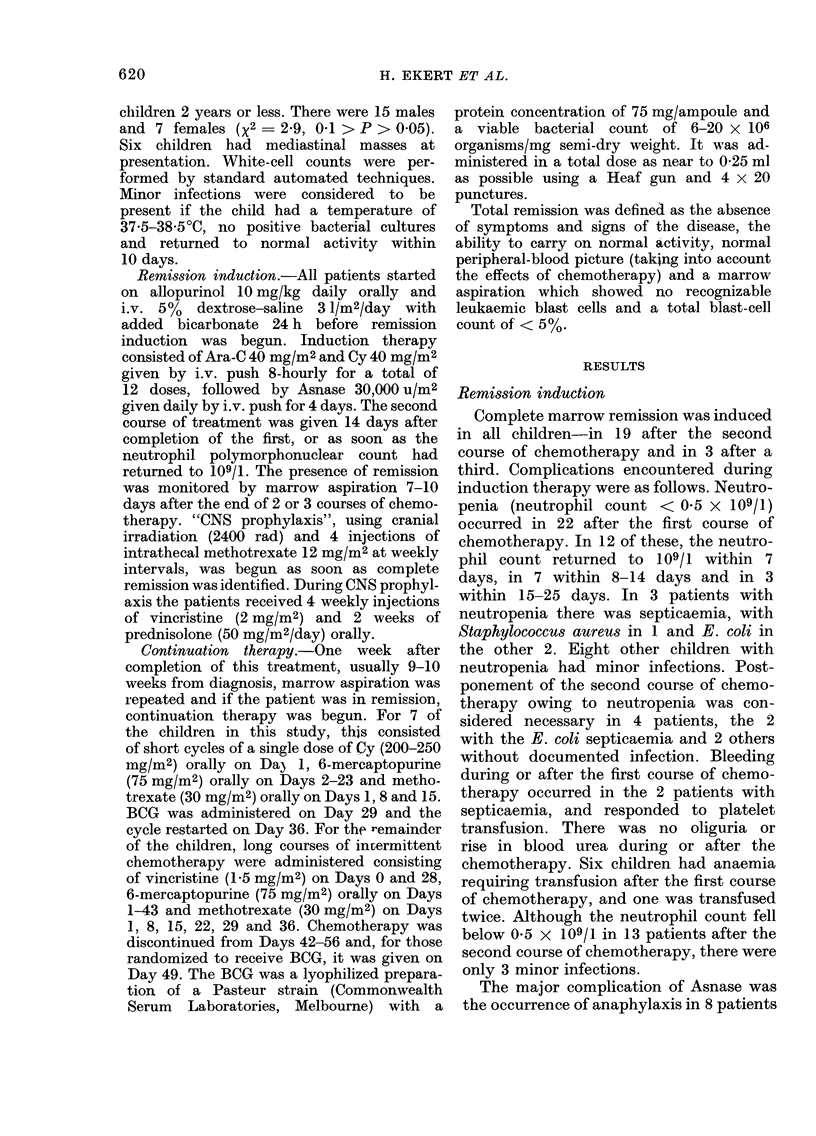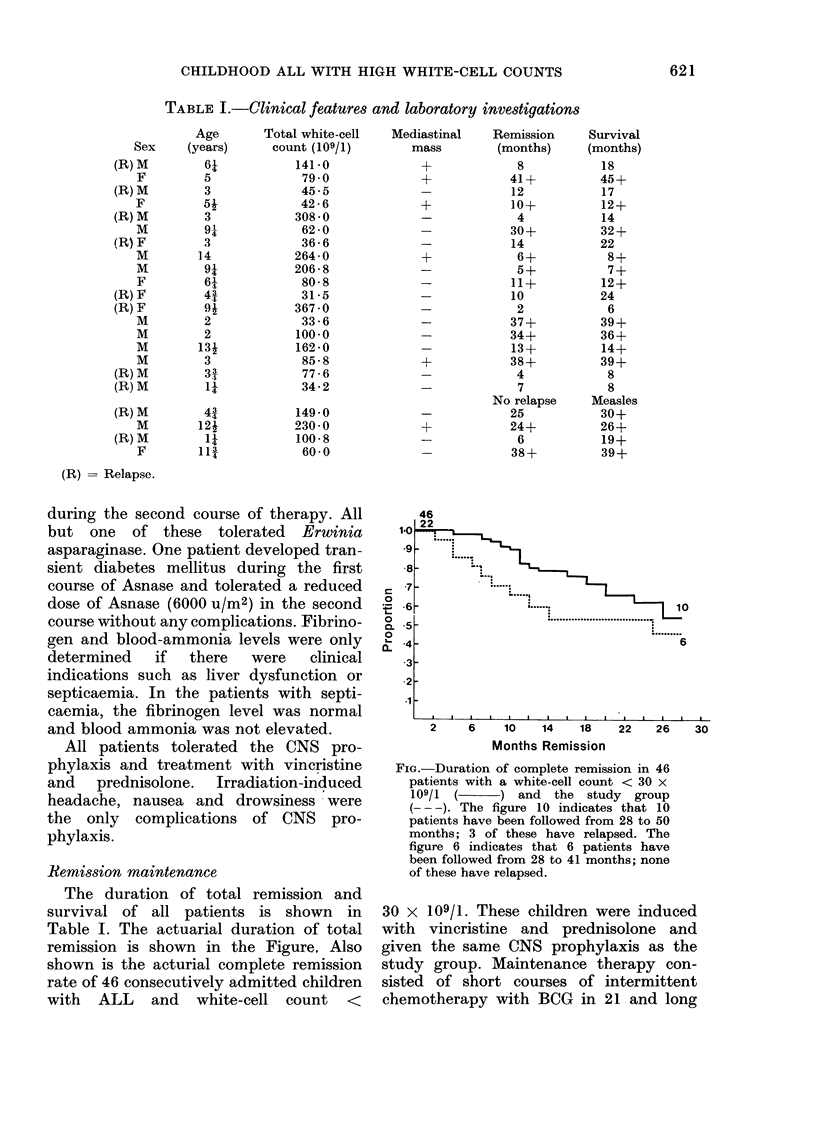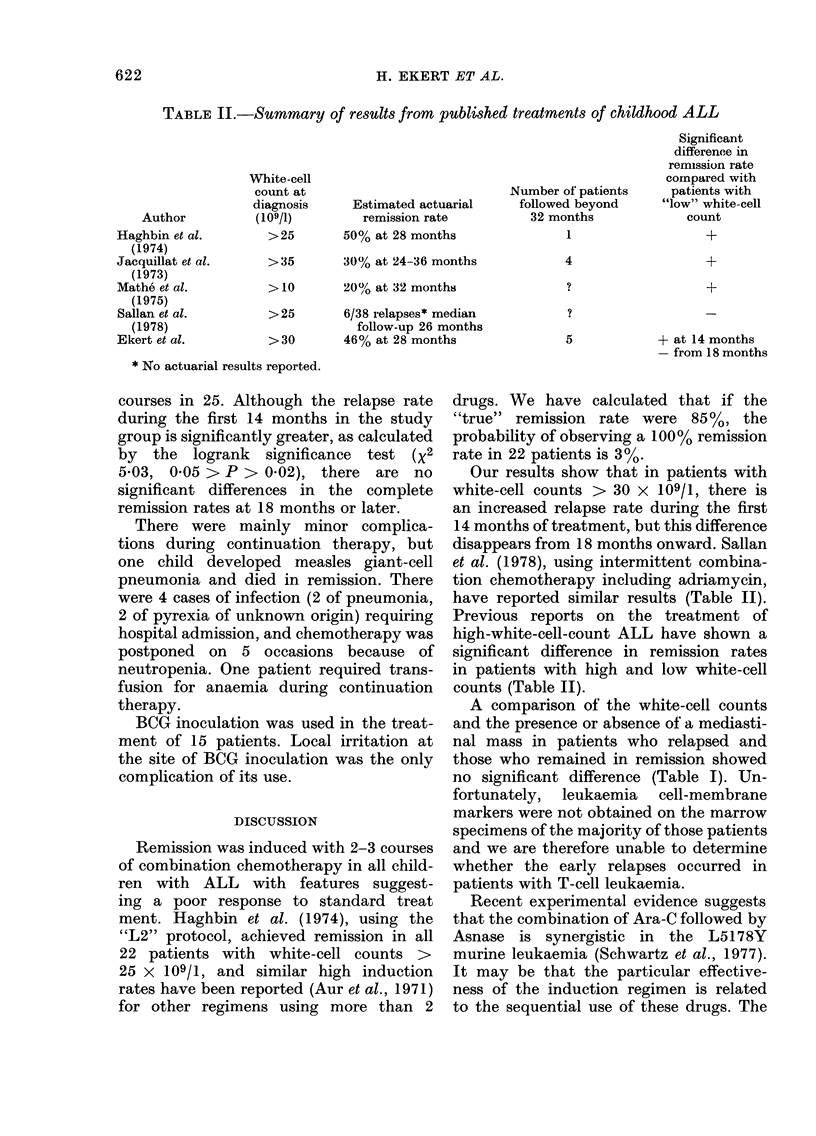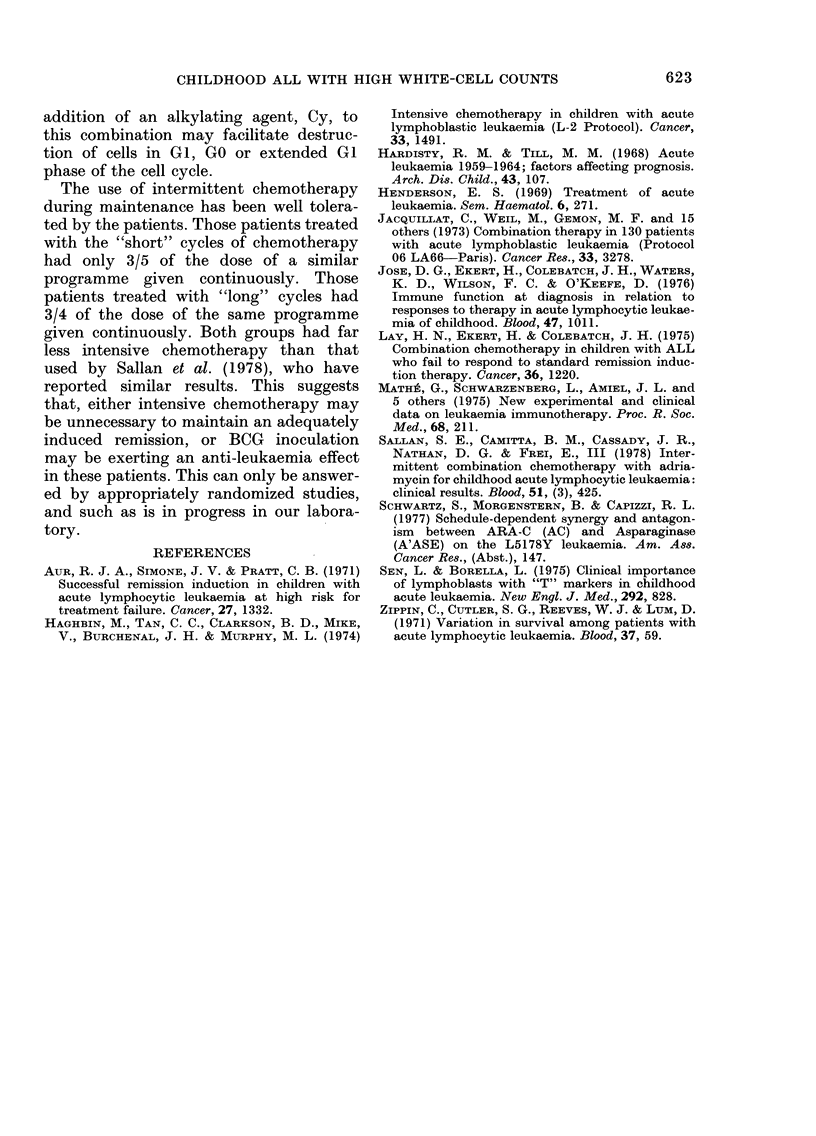# Treatment of childhood lymphocytic leukaemia with high white-cell counts.

**DOI:** 10.1038/bjc.1978.258

**Published:** 1978-11

**Authors:** H. Ekert, K. D. Waters, P. J. Smith, R. N. Matthews, W. M. Ellis

## Abstract

Combination chemotherapy with cytosine arabinoside, cyclophosphamide and L-asparaginase (Asnase) was given to 22 children with acute lymphocytic leukaemia (ALL) with a white-cell count greater than 30 X 10(9)/1, and other features suggestive of poor prognosis. Complete remission was induced in all patients--in 19 after 2 courses of chemotherapy and in the remainder after a third course. During induction, neutropenia occurred in 18 and severe infection in 3. Anaphylaxis to Asnase occurred in 8 patients after the second course and one other had transient Asnase-induced diabetes. All patients received central-nervous-system prophylaxis after achieving remission, during which they were also treated with weekly vincristine and a 2-week course of prednisolone. Continuation therapy consisted of short cycles of intermittent chemotherapy and BCG inoculation or long cycles of intermittent chemotherapy +/- BCG. Life-table analysis shows 46% complete remission rate at 28 months, with 6 patients all in complete remission followed up between 28 and 41 months. There were minimal complications of continuation therapy, and BCG inoculation was well tolerated.


*Br. J. Cancer* (1978) **38**, 619

### TREATMENT OF CHILDHOOD LYMPHOCYTIC LEUKAEMIA WITH HIGH WHITE-CELL COUNTS

H. EKERT, K. D. WATERS, P. J. SMITH, R. N. MATTHEWS AND W. M. ELLIS

*From the Department of Clinical Haematology and Oncology, Royal Children's Hospital, Parkville, Vic. 3052, Australia*

Received 8 February 1978 Accepted 9 August 1978

**Summary.—Combination chemotherapy with cytosine arabinoside, cyclophosphamide and L-asparaginase (Asnase) was given to 22 children with acute lymphocytic leukaemia (ALL) with a white-cell count greater than 30 $\times$ $10^9$/l, and other features suggestive of poor prognosis. Complete remission was induced in all patients—in 19 after 2 courses of chemotherapy and in the remainder after a third course. During induction, neutropenia occurred in 18 and severe infection in 3. Anaphylaxis to Asnase occurred in 8 patients after the second course and one other had transient Asnase-induced diabetes. All patients received central-nervous-system prophylaxis after achieving remission, during which they were also treated with weekly vincristine and a 2-week course of prednisolone. Continuation therapy consisted of short cycles of intermittent chemotherapy and BCG inoculation or long cycles of intermittent chemotherapy $\pm$ BCG. Life-table analysis shows 46% complete remission rate at 28 months, with 6 patients all in complete remission followed up between 28 and 41 months. There were minimal complications of continuation therapy, and BCG inoculation was well tolerated.**

IN childhood acute lymphocytic leukaemia (ALL) certain findings at the time of diagnosis indicate a reduced likelihood of a good response to standard treatment. These findings include: age of less than 2 or more than 12 years, a white-cell count $> 20 \times 10^9$/l, marked infiltration of liver, spleen and lymph nodes, mediastinal enlargement, and high numbers of peripheral-blood lymphocytes forming rosettes with sheep red cells at 4°C ("$T_4$") or rosette formation by marrow lymphoblasts at 37°C (Aur *et al.*, 1971; Haghbin *et al.*, 1974; Hardisty & Till, 1968; Henderson, 1969; Jose *et al.*, 1976; Sen & Borella, 1975; Zippin *et al.*, 1971).

We have previously reported that combination chemotherpay with cytosine arabinoside (Ara-C), cyclophosphamide (Cy) and *E. coli* L-asparaginase (Asnase) induced remission in children who failed to respond to standard induction programmes (Lay *et al.*, 1975). Most of these children had high white-cell counts at diagnosis. In this paper, we report the results of treatment from diagnosis of 22 children with a high white-cell count using the above drugs for induction of remission, and as continuation therapy, intermittent chemotherapy with or without BCG.

### PATIENTS AND METHODS

All children with ALL whose white-cell counts at the time of diagnosis were $> 30 \times 10^9$/l were entered into the study. Children with marked lymph-node infiltration, including the mediastinum, and who also had marrow infiltration of $> 25$% lymphoblasts were included. The relevant clinical features and laboratory investigations of the 22 children are shown in Table I. Their ages ranged from 1 to 14 years, with 4

Address for reprints: Dr H. Ekert, Director, Department of Clinical Haematology and Oncology, Royal Children's Hospital, Parkville, Vic. 3052, Australia.

children 2 years or less. There were 15 males and 7 females ($\chi^2 = 2{\cdot}9$, $0{\cdot}1 > P > 0{\cdot}05$). Six children had mediastinal masses at presentation. White-cell counts were performed by standard automated techniques. Minor infections were considered to be present if the child had a temperature of 37·5–38·5°C, no positive bacterial cultures and returned to normal activity within 10 days.

*Remission induction.*—All patients started on allopurinol 10 mg/kg daily orally and i.v. 5% dextrose–saline 3 l/m²/day with added bicarbonate 24 h before remission induction was begun. Induction therapy consisted of Ara-C 40 mg/$m^2$ and Cy 40 mg/$m^2$ given by i.v. push 8-hourly for a total of 12 doses, followed by Asnase 30,000 u/$m^2$ given daily by i.v. push for 4 days. The second course of treatment was given 14 days after completion of the first, or as soon as the neutrophil polymorphonuclear count had returned to $10^9$/l. The presence of remission was monitored by marrow aspiration 7–10 days after the end of 2 or 3 courses of chemotherapy. "CNS prophylaxis", using cranial irradiation (2400 rad) and 4 injections of intrathecal methotrexate 12 mg/$m^2$ at weekly intervals, was begun as soon as complete remission was identified. During CNS prophylaxis the patients received 4 weekly injections of vincristine (2 mg/$m^2$) and 2 weeks of prednisolone (50 mg/$m^2$/day) orally.

*Continuation therapy.*—One week after completion of this treatment, usually 9–10 weeks from diagnosis, marrow aspiration was repeated and if the patient was in remission, continuation therapy was begun. For 7 of the children in this study, this consisted of short cycles of a single dose of Cy (200–250 mg/$m^2$) orally on Day 1, 6-mercaptopurine (75 mg/$m^2$) orally on Days 2–23 and methotrexate (30 mg/$m^2$) orally on Days 1, 8 and 15. BCG was administered on Day 29 and the cycle restarted on Day 36. For the remainder of the children, long courses of intermittent chemotherapy were administered consisting of vincristine (1·5 mg/$m^2$) on Days 0 and 28, 6-mercaptopurine (75 mg/$m^2$) orally on Days 1–43 and methotrexate (30 mg/$m^2$) on Days 1, 8, 15, 22, 29 and 36. Chemotherapy was discontinued from Days 42–56 and, for those randomized to receive BCG, it was given on Day 49. The BCG was a lyophilized preparation of a Pasteur strain (Commonwealth Serum Laboratories, Melbourne) with a protein concentration of 75 mg/ampoule and a viable bacterial count of 6–20 $\times 10^6$ organisms/mg semi-dry weight. It was administered in a total dose as near to 0·25 ml as possible using a Heaf gun and 4 × 20 punctures.

Total remission was defined as the absence of symptoms and signs of the disease, the ability to carry on normal activity, normal peripheral-blood picture (taking into account the effects of chemotherapy) and a marrow aspiration which showed no recognizable leukaemic blast cells and a total blast-cell count of < 5%.

### RESULTS

### *Remission induction*

Complete marrow remission was induced in all children—in 19 after the second course of chemotherapy and in 3 after a third. Complications encountered during induction therapy were as follows. Neutropenia (neutrophil count $< 0{\cdot}5 \times 10^9$/l) occurred in 22 after the first course of chemotherapy. In 12 of these, the neutrophil count returned to $10^9$/l within 7 days, in 7 within 8–14 days and in 3 within 15–25 days. In 3 patients with neutropenia there was septicaemia, with *Staphylococcus aureus* in 1 and *E. coli* in the other 2. Eight other children with neutropenia had minor infections. Postponement of the second course of chemotherapy owing to neutropenia was considered necessary in 4 patients, the 2 with the *E. coli* septicaemia and 2 others without documented infection. Bleeding during or after the first course of chemotherapy occurred in the 2 patients with septicaemia, and responded to platelet transfusion. There was no oliguria or rise in blood urea during or after the chemotherapy. Six children had anaemia requiring transfusion after the first course of chemotherapy, and one was transfused twice. Although the neutrophil count fell below $0{\cdot}5 \times 10^9$/l in 13 patients after the second course of chemotherapy, there were only 3 minor infections.

The major complication of Asnase was the occurrence of anaphylaxis in 8 patients

TABLE I.—*Clinical features and laboratory investigations*

| Sex | Age (years) | Total white-cell count ($10^9$/1) | Mediastinal mass | Remission (months) | Survival (months) |
|---|---|---|---|---|---|
| (R) M | 6¼ | 141·0 | + | 8 | 18 |
| F | 5 | 79·0 | + | 41+ | 45+ |
| (R) M | 3 | 45·5 | — | 12 | 17 |
| F | 5½ | 42·6 | + | 10+ | 12+ |
| (R) M | 3 | 308·0 | — | 4 | 14 |
| M | 9¼ | 62·0 | — | 30+ | 32+ |
| (R) F | 3 | 36·6 | — | 14 | 22 |
| M | 14 | 264·0 | + | 6+ | 8+ |
| M | 9¼ | 206·8 | — | 5+ | 7+ |
| F | 6¼ | 80·8 | — | 11+ | 12+ |
| (R) F | 4¾ | 31·5 | — | 10 | 24 |
| (R) F | 9½ | 367·0 | — | 2 | 6 |
| M | 2 | 33·6 | — | 37+ | 39+ |
| M | 2 | 100·0 | — | 34+ | 36+ |
| M | 13½ | 162·0 | — | 13+ | 14+ |
| M | 3 | 85·8 | + | 38+ | 39+ |
| (R) M | 3¾ | 77·6 | — | 4 | 8 |
| (R) M | 1¼ | 34·2 | — | 7 | 8 |
| | | | | No relapse | Measles |
| (R) M | 4¾ | 149·0 | — | 25 | 30+ |
| M | 12½ | 230·0 | + | 24+ | 26+ |
| (R) M | 1¼ | 100·8 | — | 6 | 19+ |
| F | 11¾ | 60·0 | — | 38+ | 39+ |

(R) = Relapse.

during the second course of therapy. All but one of these tolerated *Erwinia* asparaginase. One patient developed transient diabetes mellitus during the first course of Asnase and tolerated a reduced dose of Asnase (6000 u/m$^2$) in the second course without any complications. Fibrinogen and blood-ammonia levels were only determined if there were clinical indications such as liver dysfunction or septicaemia. In the patients with septicaemia, the fibrinogen level was normal and blood ammonia was not elevated.

All patients tolerated the CNS prophylaxis and treatment with vincristine and prednisolone. Irradiation-induced headache, nausea and drowsiness were the only complications of CNS prophylaxis.

*Remission maintenance*

The duration of total remission and survival of all patients is shown in Table I. The actuarial duration of total remission is shown in the Figure. Also shown is the acturial complete remission rate of 46 consecutively admitted children with ALL and white-cell count < 30 × $10^9$/1. These children were induced with vincristine and prednisolone and given the same CNS prophylaxis as the study group. Maintenance therapy consisted of short courses of intermittent chemotherapy with BCG in 21 and long

FIG.—Duration of complete remission in 46 patients with a white-cell count < 30 × $10^9$/1 (———) and the study group (- - -). The figure 10 indicates that 10 patients have been followed from 28 to 50 months; 3 of these have relapsed. The figure 6 indicates that 6 patients have been followed from 28 to 41 months; none of these have relapsed.

TABLE II.—*Summary of results from published treatments of childhood ALL*

| Author | White-cell count at diagnosis ($10^9$/l) | Estimated actuarial remission rate | Number of patients followed beyond 32 months | Significant difference in remission rate compared with patients with "low" white-cell count |
|---|---|---|---|---|
| Haghbin *et al.* (1974) | >25 | 50% at 28 months | 1 | + |
| Jacquillat *et al.* (1973) | >35 | 30% at 24–36 months | 4 | + |
| Mathé *et al.* (1975) | >10 | 20% at 32 months | ? | + |
| Sallan *et al.* (1978) | >25 | 6/38 relapses* median follow-up 26 months | ? | — |
| Ekert *et al.* | >30 | 46% at 28 months | 5 | + at 14 months<br>— from 18 months |

* No actuarial results reported.

courses in 25. Although the relapse rate during the first 14 months in the study group is significantly greater, as calculated by the logrank significance test ($\chi^2$ 5·03, $0{\cdot}05 > P > 0{\cdot}02$), there are no significant differences in the complete remission rates at 18 months or later.

There were mainly minor complications during continuation therapy, but one child developed measles giant-cell pneumonia and died in remission. There were 4 cases of infection (2 of pneumonia, 2 of pyrexia of unknown origin) requiring hospital admission, and chemotherapy was postponed on 5 occasions because of neutropenia. One patient required transfusion for anaemia during continuation therapy.

BCG inoculation was used in the treatment of 15 patients. Local irritation at the site of BCG inoculation was the only complication of its use.

### DISCUSSION

Remission was induced with 2–3 courses of combination chemotherapy in all children with ALL with features suggesting a poor response to standard treat ment. Haghbin *et al.* (1974), using the "L2" protocol, achieved remission in all 22 patients with white-cell counts > $25 \times 10^9$/l, and similar high induction rates have been reported (Aur *et al.*, 1971) for other regimens using more than 2 drugs. We have calculated that if the "true" remission rate were 85%, the probability of observing a 100% remission rate in 22 patients is 3%.

Our results show that in patients with white-cell counts > $30 \times 10^9$/l, there is an increased relapse rate during the first 14 months of treatment, but this difference disappears from 18 months onward. Sallan *et al.* (1978), using intermittent combination chemotherapy including adriamycin, have reported similar results (Table II). Previous reports on the treatment of high-white-cell-count ALL have shown a significant difference in remission rates in patients with high and low white-cell counts (Table II).

A comparison of the white-cell counts and the presence or absence of a mediastinal mass in patients who relapsed and those who remained in remission showed no significant difference (Table I). Unfortunately, leukaemia cell-membrane markers were not obtained on the marrow specimens of the majority of those patients and we are therefore unable to determine whether the early relapses occurred in patients with T-cell leukaemia.

Recent experimental evidence suggests that the combination of Ara-C followed by Asnase is synergistic in the L5178Y murine leukaemia (Schwartz *et al.*, 1977). It may be that the particular effectiveness of the induction regimen is related to the sequential use of these drugs. The addition of an alkylating agent, Cy, to this combination may facilitate destruction of cells in G1, G0 or extended G1 phase of the cell cycle.

The use of intermittent chemotherapy during maintenance has been well tolerated by the patients. Those patients treated with the "short" cycles of chemotherapy had only 3/5 of the dose of a similar programme given continuously. Those patients treated with "long" cycles had 3/4 of the dose of the same programme given continuously. Both groups had far less intensive chemotherapy than that used by Sallan *et al.* (1978), who have reported similar results. This suggests that, either intensive chemotherapy may be unnecessary to maintain an adequately induced remission, or BCG inoculation may be exerting an anti-leukaemia effect in these patients. This can only be answered by appropriately randomized studies, and such as is in progress in our laboratory.

### REFERENCES

Aur, R. J. A., Simone, J. V. & Pratt, C. B. (1971) Successful remission induction in children with acute lymphocytic leukaemia at high risk for treatment failure. *Cancer*, **27**, 1332.

Haghbin, M., Tan, C. C., Clarkson, B. D., Mike, V., Burchenal, J. H. & Murphy, M. L. (1974) Intensive chemotherapy in children with acute lymphoblastic leukaemia (L-2 Protocol). *Cancer*, **33**, 1491.

Hardisty, R. M. & Till, M. M. (1968) Acute leukaemia 1959–1964; factors affecting prognosis. *Arch. Dis. Child.*, **43**, 107.

Henderson, E. S. (1969) Treatment of acute leukaemia. *Sem. Haematol.* **6**, 271.

Jacquillat, C., Weil, M., Gemon, M. F. and 15 others (1973) Combination therapy in 130 patients with acute lymphoblastic leukaemia (Protocol 06 LA66—Paris). *Cancer Res.*, **33**, 3278.

Jose, D. G., Ekert, H., Colebatch, J. H., Waters, K. D., Wilson, F. C. & O'Keefe, D. (1976) Immune function at diagnosis in relation to responses to therapy in acute lymphocytic leukaemia of childhood. *Blood*, **47**, 1011.

Lay, H. N., Ekert, H. & Colebatch, J. H. (1975) Combination chemotherapy in children with ALL who fail to respond to standard remission induction therapy. *Cancer*, **36**, 1220.

Mathé, G., Schwarzenberg, L., Amiel, J. L. and 5 others (1975) New experimental and clinical data on leukaemia immunotherapy. *Proc. R. Soc. Med.*, **68**, 211.

Sallan, S. E., Camitta, B. M., Cassady, J. R., Nathan, D. G. & Frei, E., III (1978) Intermittent combination chemotherapy with adriamycin for childhood acute lymphocytic leukaemia: clinical results. *Blood*, **51**, (3), 425.

Schwartz, S., Morgenstern, B. & Capizzi, R. L. (1977) Schedule-dependent synergy and antagonism between ARA-C (AC) and Asparaginase (A'ASE) on the L5178Y leukaemia. *Am. Ass. Cancer Res.*, (Abst.), 147.

Sen, L. & Borella, L. (1975) Clinical importance of lymphoblasts with "T" markers in childhood acute leukaemia. *New Engl. J. Med.*, **292**, 828.

Zippin, C., Cutler, S. G., Reeves, W. J. & Lum, D. (1971) Variation in survival among patients with acute lymphocytic leukaemia. *Blood*, **37**, 59.